# Ulcer of the Stomach and Duodenum

**Published:** 1900-12

**Authors:** 


**Ulcer of the Stomach and Duodenum.** By Samuel Fenwick, M.D., and W. Soltau Fenwick, M.D. Pp. xiv., 392. London: J. & A. Churchill. 1900.

This is an exhaustive book, and, like all books which contain records of cases, ought to be of lasting value. The difficult question of the ætiology of gastric ulcer is fully discussed. The authors consider that acute primary ulcer often commences as a hemorrhage, and that chronic ulcer, although it may be a sequel of an acute ulcer, is probably frequently due to impaired nutrition of the mucous membrane of the stomach consequent upon arterial degeneration. It has always seemed to us that a hemorrhage is the most likely cause of a gastric ulcer, when it occurs in an anæmic girl with cardiac failure consequent upon the anæmia. It seems to us, however, that micro-organisms probably play an important part in preventing healing of the damaged mucous membrane. That micro-organisms of infective character are commonly present in the base of an ulcer is obvious, from the frequency with which the wall of the stomach opposite to an ulcer becomes attacked, and a second ulcer produced. The situation of an acute ulcer near the lesser curvature of the stomach, where it is not continually bathed with bactericidal gastric juice, also suggests that micro-organisms are an important factor in maintaining ulceration.

This volume enters fully into the symptomatology and treatment of gastric ulcer. When speaking of the symptoms, it is mentioned that pain after food occurs in 21 per cent. of acute primary gastric ulcer, but the interesting statement is made that "only about seven per cent. of the cases of this dangerous disease complain of that severe form of pain which is supposed to be characteristic of gastric ulcer." The diagnosis of chronic ulcer is discussed under the headings of gastralgic, catarrhal or vomiting, dyspeptic, hemorrhagic and cachectic forms.

The numerous complications of gastric ulcer, of which perhaps the most important is subphrenic abscess, are also fully dealt with.

